# Limitations of Fixed-Point-Charge Force Fields for Metal Coordination Modes in Proteins: A Multilevel Study of Ca^2+^ in Integrin α_V_β_3_

**DOI:** 10.1021/acs.jctc.6c01320

**Published:** 2026-10-28

**Authors:** Andrea Levy, Giulia Frigerio, Jules Grollier, Paulo Siani, Cristiana Di Valentin, Ursula Rothlisberger

**Affiliations:** † Laboratory of Computational Chemistry and Biochemistry, École Polytechnique Fédérale de Lausanne, Lausanne, CH-1015, Switzerland; ‡ Department of Materials Science, 9305 University of Milano-Bicocca, Via R. Cozzi 55, 20125 Milan, Italy; § BioNanoMedicine Center NANOMIB, University of Milano-Bicocca, Milan, 20125 Italy

## Abstract

Standard biomolecular force fields often present limitations in modeling metal coordination modes. We combined classical and QM/MM molecular dynamics to investigate Ca^2+^-mediated binding of cRGD to integrin α_V_β_3_. We show that different metal coordination modes imply variations in the partial charges of the metal-coordinating atoms, which, if incorporated in the force field, substantially alter the free energy landscape. These results highlight the value of QM-based multilevel approaches to accurately capture metal coordination modes in biomolecular systems.

## Graphical abstract

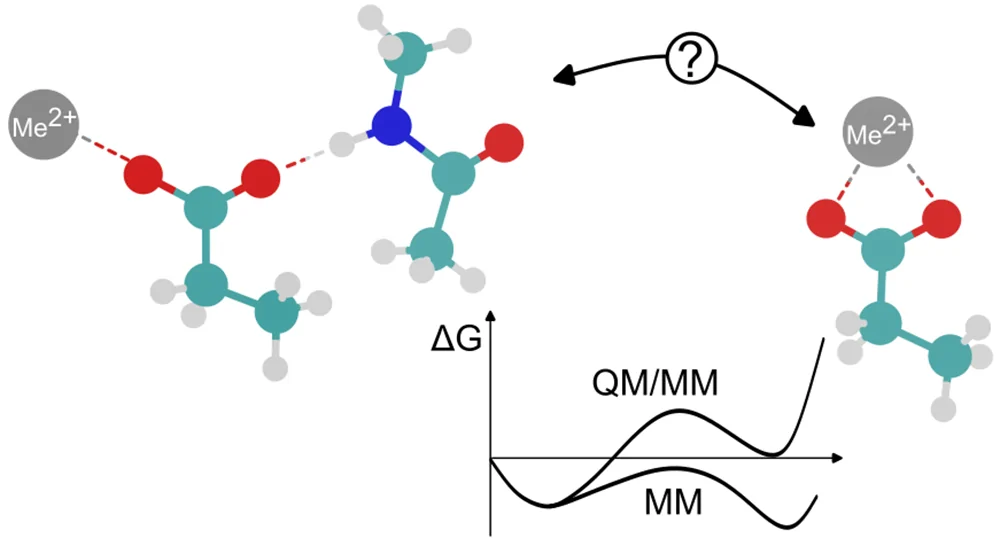

## 1 Introduction

Molecular dynamics (MD) simulations based on classical fixed-point-charge atomistic force fields (FFs) are a vital tool in the field of computational chemistry.^1–11^ By describing interatomic interactions through pairwise additive potentials, these methods enable simulations of systems containing several hundred thousands of atoms, reaching microsecond time scales at a reasonable computational cost.^12–16^ This has led to remarkable successes in biomolecular simulations.^17–19^ However, the functional forms of standard biomolecular FFs impose limitations in the description of phenomena such as charge transfer and polarization effects,^20–23^ metal coordination,^24–27^ or cation-π interactions.^28–30^ In particular, metal coordination modes involve a complex interplay between electrostatics, polarization, charge transfer, and directionality imposed by the electronic structure of the metal ion and its ligands, as well as the competition between different ligands. Therefore, metal coordination modes are especially challenging to model with fixed-point-charge FFs.^31^

Because of these limitations, it is often necessary to increase the level of theory for the whole or for part of the system to ensure that there are no artifacts due to the FF description. For example, it is possible to partition the system into a small region treated at the quantum-mechanical (QM) level, with the remainder described at the molecular mechanics (MM) level, using fixed-point-charge FFs. In this hybrid QM/MM approach, the electronic structure of the chemically active region, such as metal coordination sites, is treated explicitly while taking into account the effects of the surrounding environment at a less computationally demanding level. This multiscale approach is particularly relevant for biological systems containing metal ions, where an accurate description of the metal coordination modes is crucial.

Notably, approximately 33% of protein structures deposited in the Protein Databank (PDB) contain metal ions.^32^ Among these, integrins represent a large family of cell-surface receptors mediating crucial interactions between cells and the extracellular matrix.^33^ Integrin α_V_β_3_ is one of the most important and clinically relevant members of this family, as it is overexpressed in several cancer types and widely targeted for both active drug delivery^34^ and anticancer therapy (e.g., cilengitide for glioblastoma^35^). Due to the large size of this protein, totaling more than 1600 amino acids, most ligand and drug design studies in the literature have relied on fixed-point-charge FFs.^36–40^ However, integrin α_V_β_3_ provides a relevant and challenging test case for fixed-point-charge FFs, as its ligand-binding site contains a divalent cation at the so-called Metal Ion–Dependent Adhesion Site (MIDAS). In addition, two other divalent cation sites are located near the binding pocket, namely the Adjacent to MIDAS (ADMIDAS) ion, more exposed to the solvent, and the Ligand-Induced Metal Binding Site (LIMBS), buried in the protein. The MIDAS ion directly mediates ligand recognition by coordinating the carboxylate group of the Asp residue within the (Arg-Gly-Asp) RGD motif, which is the amino acidic sequence selectively recognized by integrin α_V_β_3_ both in natural and synthetic ligands.^34,41,42^ Experimental X-ray structures reveal a *bridging* coordination mode (Figure 1), where one of the O atoms of the RGD Asp carboxylate is pointing toward the MIDAS ion and the other one to the integrin backbone.^43^ In contrast, MD simulations performed at the molecular mechanics (MM) level with the fixed-point-charge FF CHARMM36m/CGenFF^5–7,44–46^ by Frigerio et al.^47,48^ provide a dynamic picture in which the binding mode interconverts between the *bridging* coordination mode and the *chelating* one, where both O atoms coordinate the MIDAS ion (Figure 1). These discrepancies between experimental and computational observations leave it unclear whether the *chelating* coordination mode observed in MM MD simulations reflects a realistic conformation accessible under aqueous biological conditions that is not captured in the crystalline state of the X-ray structures, or whether it represents an artifact of the fixed-point-charge FF. In this work, we present a detailed investigation of the metal coordination modes in the integrin α_V_β_3_/ligand (cyclic RGD, c­(RGDyK)—hereafter referred to as cRGD) complex, focusing on the MIDAS ion. By using a higher level of theoretical description, we aim to determine if the metal coordination modes observed in fixed-point-charge FF-based MD simulations likely correspond to physically relevant states or to FF artifacts. Starting from classical FF results, we employ QM/MM MD to assess the stability of the two metal coordination modes. Although the current study focuses on a single system treated with a specific FF, the results and conclusions highlight some inherent universal possible issues of fixed-point-charge FFs in modeling metal coordination modes in biological systems and illustrate how QM/MM can be used to validate and potentially improve the FF description.

**Figure 1:**
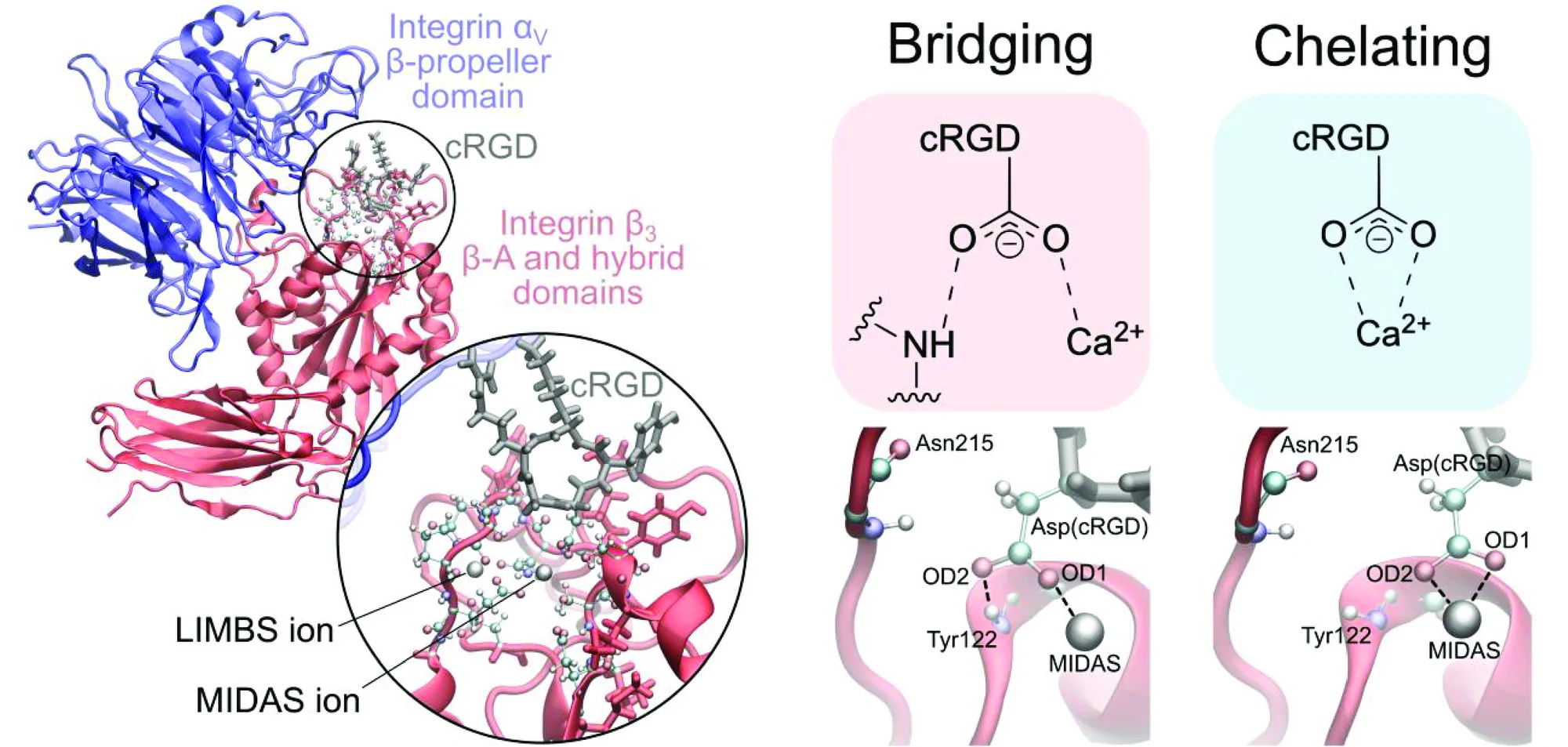
Model system used for MM and QM/MM MD simulations (left). The QM region (indicated with the circles) highlights the atoms treated at QM level in ball and stick representation (water molecules are omitted for clarity). Metal coordination modes for MIDAS ion (right). For both modes, a schematic representation is reported, as well as the initial configuration for the unconstrained QM/MM MD, where only the relevant residues are represented.

In the following, we discuss the systems and simulation protocols in Section 2. We present the results for the integrin system in Section 3, as well as the impact of refining the CHARMM36m/CGenFF description. Finally, in Section 4, starting from summarizing our conclusions, we discuss more general implications for modeling metal coordination modes in biological systems using classical FFs based on fixed point charges.

## 2 Computational Details

The model system is composed by the headpiece of integrin α_V_β_3_ in complex with the cRGD ligand, which shares the cyclic structure characteristic of typical integrin α_V_β_3_-selective ligands.^36,38^

The structure of the integrin α_V_β_3_ extracellular segment was taken from the PDB (code 4MMX).^49^ The fibronectin ligand was removed while the two cocrystallized waters in the binding pocket were kept and the eight cocrystallized metal ions were modeled as Ca^2+^ ions, according to a common practice.^38,50,51^ The protein structure was processed with the *Protein Preparation Wizard* in Maestro (release 2021–1).^52^ The cRGD ligand was docked into the binding site with the rigid receptor docking protocol described in the SI, using the *Glide* software.^53–55^ To reduce the computational cost of MD simulations, we considered only the β-propeller domain for chain α and β-A and the hybrid domains for chain β, which constitute the headpiece of integrin α_V_β_3_ containing the ligand binding site and seven structural Ca^2+^. This strategy was adopted also in other works.^56–60^

The protein/ligand complex was prepared for MM MD simulations using the *Ligand Reader & Modeler* CHARMM-GUI module to generate topology and FF parameters.^61^ The same FF as in the previous work by some of us^47^ was used: CHARMM36m FF^5,6,44^ for the protein and CGenFF^7,45,46^ for the cRGD ligand, removing the NBFIX correction for Ca^2+^ cations, whose impact has been assessed in ref 47. The system was solvated in an aqueous solution of NaCl (0.15M), using the rigid CHARMM-modified TIP3P model^5,62^ in a cubic box with a side length of 125Å, with GROMACS tools^63^ or CHARMM-GUI *Solution Builder* module,^61,64^ to obtain independent replicas with different initial solvent configurations. Three independent replicas were simulated with different initial setups: one 1 μs simulation starting from the system solvated with GROMACS tools, and two 500 ns simulations both starting from the system solvated with the CHARMM-GUI *Solution Builder* and differing in their initial velocity assignments. For each replica, a minimization of 5000 steps using the steepest descent algorithm was carried out, followed by a 2 ns NVT equilibration and three NPT runs. The temperature was set at 303.15K by V-Rescale thermostat,^65^ with a coupling constant of 1.0 ps. The pressure was kept constant at 1 bar by a Parrinello–Rahman barostat^66^ with a coupling constant of 5.0 ps, with fixed box angles and only the box lengths allowed to vary. The Particle Mesh Ewald (PME) solver^67^ handled the electrostatic interactions with a real-space cutoff of 12.0 Å, while van der Waals interactions were smoothly switched to zero using a force-switch function between an inner cutoff of 10 Å and an outer cutoff of 12 Å. Newton’s equations of motion were integrated with the leapfrog algorithm.^68^ All covalent bonds involving a hydrogen atom were constrained using LINCS.^63,69^ MM MD simulations were performed using GROMACS.^63^

QM/MM MD simulations were performed using the MiMiC framework,^70,71^ which assigns different external programs to different subsystems. The QM subsystem (Figure 1) is composed of 247 atoms at the binding site of the cRGD ligand and contains the MIDAS ion, as well as the nearby LIMBS ion with their coordinating residues and 33 water molecules chosen based on the distance from the MIDAS ion (all water molecules with at least one atom within 9 Å of the MIDAS ion in the starting configuration). The QM subsystem was handled by CP2K^72,73^ and was treated at DFT level with the BLYP functional^74,75^ with density and relative cutoffs (750 and 90 Ry, respectively) determined using standard procedures in CP2K,^73^ using a cubic box of length 40 Å and the multipole Poisson solver available in CP2K to decouple QM periodic images with the use of density derived atomic point charges.^76^ The MM subsystem was handled by GROMACS,^63,77,78^ with the same parameters as in the previous MM MD simulations. We also converged the QM/MM parameters for the long-/short-range QM/MM electrostatic coupling in MiMiC according to ref 71, resulting in a short-range cutoff of 80*a*_0_ (∼42 Å) and a multipole order of 7 for the expansion of the QM density. The MD propagation (Born–Oppenheimer MD) was handled by the MD driver, CPMD,^79^ using a time step of 20ℏ/*E_h_* (∼0.5 fs) and Nosé-Hoover thermostats^80,81^ on the two subsystem (with a reference temperature of 303.15 K and coupling frequency 3000 cm^–1^).

In the QM/MM thermodynamic integration (TI)^82–84^ simulations, the MD driver handled the constraints, and a distance constraint was imposed between the MIDAS ion and the OD2 oxygen of the cRGD ligand. Each TI window was simulated for around 1 ps until the average constraint force reached convergence (Figures S4–S8). Configurations from the QM/MM TI windows were used as starting points for the MM TI, in which similar constraints were imposed with GROMACS. In this case, each window was simulated for longer time scales (around 1 ns) until the convergence of the average constraint force.

## 3 Results and Discussion

### 3.1 MM MD Simulations

Our classical MD simulations of cRGD in complex with integrin α_V_β_3_, including a 1 μs simulation (Figure S2) and two 500 ns replicas (Figure S3) as detailed in Section 2, consistently reveal two distinct coordination modes of the MIDAS ion with the cRGD Asp carboxylate: *bridging* and *chelating* (Figure 1), which were also found in a previous work by some of us.^47^ In particular, in the *bridging* mode, one carboxylate oxygen—hereafter referred to as OD1—coordinates the MIDAS Ca^2+^ ion in a monodentate fashion (distance Ca–OD1 around 2.0–2.5 Å), while the second oxygen—hereafter referred to as OD2—forms a hydrogen bond with the backbone amide of Tyr122 in the β chain (distance Ca–OD2 around 4.0 Å). In the *chelating* mode on the other hand, both carboxylate oxygens directly coordinate the metal ion (both distances Ca–OD1 and Ca–OD2 around 2.0–2.5 Å). We also calculated the coordination number of the Ca^2+^ ion, both as a total and separately for each contributing species (protein, cRGD, and water), using a 2.5 Å distance cutoff and considering only oxygen atoms, as these are the sole atoms directly coordinating the cation. This cutoff was chosen based on the average Ca–OD2 distance, considering values within one standard deviation of the mean for the *chelating* mode (Table 1). In the MM MD, the total coordination number fluctuates mainly between 5 and 7 (Figure S13) with average values of 6.0 ± 0.1 and 6.8 ± 0.4 for the bridging and the *chelating* mode, respectively (Table 1). As expected, the difference between the *chelating* and the *bridging* modes comes from the cRGD contribution (correctly reproduced by the FF), but the deviation between the FF and QM/MM lies in the coordination to the protein.

**Table 1:** Distances between OD1 and OD2 Oxygen Atoms and the MIDAS Ca^2+^ Ion and Its Coordination Number (Quantified as the Number of Coordinating Oxygen Atoms) during the Unconstrained MM and QM/MM MD^a^

Simulation	MM MD^b^		QM/MM MD	
Coordination mode	*Bridging*	*Chelating*	*Bridging*	*Chelating*
Ca–OD1 distance (Å)	2.10 ± 0.04	2.2 ± 0.1	2.3 ± 0.1	2.5 ± 0.1
Ca–OD2 distance (Å)	4.0 ± 0.1	2.3 ± 0.2	4.2 ± 0.1	2.6 ± 0.2
Coordinating oxygen atoms (Total)	6.0 ± 0.1	6.8 ± 0.4	5.0 ± 0.1	5.9 ± 0.4
Coordinating oxygen atoms (Protein)	3.0 ± 0.1	2.9 ± 0.3	2.0 ± 0.1	2.0 ± 0.0
Coordinating oxygen atoms (Water)	2.00 ± 0.04	2.0 ± 0.2	2.0 ± 0.1	2.1 ± 0.3
Coordinating oxygen atoms (cRGD)	1.0 ± 0.0	1.9 ± 0.3	1.0 ± 0.0	1.9 ± 0.3

a For each value, the standard deviation is also reported.

b 1 μs replica, bridging (Ca–OD2 > 3.5 Å) and chelating (Ca–OD2 ≤ 3.5 Å)

We note that the transition between *bridging* and *chelating* modes occurs at different times in the 1 μs and the 500 ns replicas, with transient intermediate states observed during the interconversion in the latter case. This variability is consistent with the interconversion being an activated process, for which the transition time is not expected to occur on the exact same time scale even for a constant barrier height, but rather follows a distribution. Variability between a few independent trajectories is therefore expected. The average Ca–OD1/OD2 distances for each binding mode reported in Table 1 were collected over the 1 μs classical MD, where no intermediate states were observed.

### 3.2 QM/MM MD Simulations

In order to perform QM/MM MD simulations, we extracted two representative well-equilibrated configurations from the MM MD simulations based on visual inspection, where the system is in the *bridging* (at 500 ns of the 1 μs replica) or in the *chelating* binding mode (at the end of the 1 μs replica). For both cases, we performed a typical QM/MM equilibration to relax the system at the QM/MM level: after a simulated annealing phase to reduce the energy of the QM region, dynamically, letting the MM region adapt, the system was slowly heated to reach the desired temperature of 303.15K, to match the one used for the classical MD.

After this equilibration, NVT QM/MM MD simulations have been performed for ∼10 ps each, and both coordination binding modes were maintained through the MD, with the average Ca–OD1/OD2 distances reported in Table 1. The evolution of these distances during the QM/MM MD is reported in the SI (Figures S9 and S10).

To ensure that no MM water replaced a QM water in the MIDAS ion coordination shell, we monitored the minimum distance between MM waters and the MIDAS ion throughout the unconstrained simulations, finding average values of 6.4 ± 0.4 Å and 7.1 ± 0.4 Å for the *bridging* and *chelating* modes, respectively (see Figure S12 for additional details). We performed an analogous analysis of MIDAS ion coordination as previously done for the MM MD case, using a 2.8 Å distance cutoff in this case to reflect the larger Ca–OD2 distance observed at this level of theory (Table 1, Figures S14 and S15). The MIDAS ion is coordinated by two waters and two protein oxygen atoms in both modes, and by one or two oxygen atoms of cRGD in the *bridging* and *chelating* modes, respectively. Notably, the main difference with respect to the MM MD results is the coordination with the protein oxygen atoms (two in the QM/MM case, and three in the MM case). This is due to the side chain of Ser123 whose distance from the cation increases in the QM/MM MD (Ca–O_Ser_: 4.6 ± 0.4 Å and 4.3 ± 0.3 Å for the *chelating* and *bridging* modes, respectively) with respect to the MM MD (Ca–O_Ser_: 2.32 ± 0.07 Å and 2.30 ± 0.07 Å for the *chelating* and *bridging* modes, respectively). Nevertheless, within a given level of description, the coordination with the protein is consistent for the two binding modes, and the only difference in the total coordination number comes from the cRGD.

Notably, the equilibrium Ca–OD1/OD2 distances and coordination numbers differ systematically between the MM and QM/MM descriptions (Table 1). As the QM/MM MD simulations were initiated from MM-equilibrated conformations and explored only a comparatively short time scale without large-scale rearrangement, these structural changes are mainly driven by the change in the level of theory, although some contribution from conformational sampling cannot be excluded.

Starting from the *chelating* binding mode, intermediate configurations have been generated by systematically varying distance constraints, in order to increase the Ca–OD2 distance and to obtain starting configurations along the reaction path from the *chelating* to the *bridging* mode. Such configurations were used to perform TI,^82–84^ where constrained QM/MM MD simulations are performed along the reaction path (collective variable, CV = Ca–OD2 distance). By integrating the average constraint force along the CV, we obtained the free energy profile reported in Figure 2, where results from the unconstrained QM/MM MD simulations have been used to add two “TI windows” with zero constraint force, in which the system is stable without the need of imposing any additional constraints.

**Figure 2:**
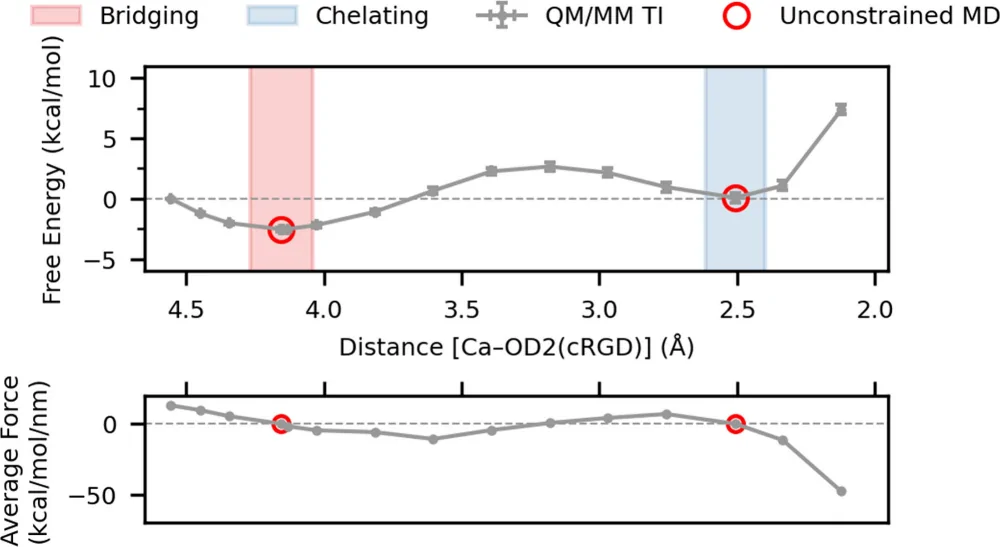
Free energy profile from QM/MM TI (upper panel) with corresponding average constraint forces (lower panel). Unconstrained QM/MM MD simulations have been used to add zero-force points at the average unconstrained distance. For each unconstrained simulation, the standard deviation of the distance is also indicated in the plot as a colored area.

From the results of QM/MM simulations, the two different coordinating modes appear to be both stable minima for cRGD, but among them the *bridging* binding mode, which is also the one observed in experimentally determined crystallographic structures,^43^ has a free energy ∼2.6 kcal/mol lower. Moreover, the two coordination minima are separated by a free energy barrier of ∼5.2 kcal/mol (*bridging* → *chelating*) and ∼2.6 kcal/mol (*chelating* → *bridging*). These barriers correspond to crossing times of ∼1 ns and ∼10 ps, respectively (estimated through the Eyring–Polanyi equation^85^ assuming zero recrossings and a constant prefactor).

### 3.3 Atomic Charge Analysis

During the QM/MM MD simulations, we made use of the xDRESP method, recently introduced within the MiMiC framework,^86^ in which the electrostatic potential generated from the QM subsystem on the surrounding MM atoms is used for determining atom-centered electric multipoles. The results of this analysis can be used to have a qualitative understanding of how the electron density is affected during a reaction. Notably, in our case, the atomic charge of the MIDAS ion remains stable during the unconstrained MD in both the *bridging* (0.47 ± 0.03 *e*) and the *chelating* (0.48 ± 0.03 *e*) conformation (Figure 3). In both cases, its charge is lower than the formal oxidation state of Ca^2+^, which is a general phenomenon for different charge schemes because electron-donating ligands, such as the carboxylic group in this case, transfer part of their negative charge to the metal ion. The xDRESP charges can be even lower than those obtained with other charge schemes, as they use Hirshfeld charges^87^ as restraints during the fit which are based on the isolated-atom densities (neutral). Nevertheless, the xDRESP point charges of the two oxygen atoms of the cRGD ligand, involved in the *chelating* or *bridging* mode, present larger differences in the two metal coordination modes, especially in the case of the OD1 oxygen, which is the one bound to the cation in both configurations and whose charge changes from −0.42 ± 0.05 *e* in the *bridging* mode to −0.28 ± 0.04 *e* in the *chelating* one (Figure 3).

**Figure 3:**
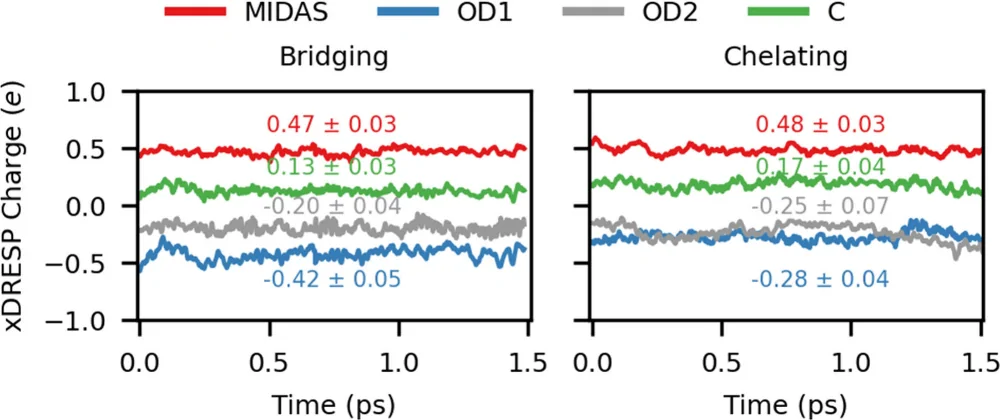
xDRESP charges from the last ∼1.5 ps of the unconstrained QM/MM MD for the two coordination modes. For both cases, the xDRESP charge of the MIDAS ion (red), OD1 oxygen (blue), OD2 oxygen (gray) and carboxylate carbon (green) are reported. The corresponding average values and standard deviations are also reported using the same color code.

In comparison, the FF used during the MM MD simulations assigns the same charge (−0.776 *e*) for the two oxygens, as they are chemically equivalent in the isolated cRGD ligand (Table 2). To further investigate the dependence of the point charges from the coordination mode, we performed RESP fitting on two model systems designed to mimic the chemical environment of the two coordination modes (Figure S11), where we constrained all the point charges—except the ones of the two oxygen atoms—to their value in the original FF. In particular, as the MM model uses the CGenFF,^45,46^ in which initial guess partial atomic charges are estimated from MP2/6–31G­(d) Merz–Kollman charges,^88,89^ we performed geometry optimizations of the model systems using the same level of theory (using Gaussian16 ^90^), and Multiwfn for the RESP fit.^91,92^

**Table 2:** Effective Atomic Charges for the OD1 and OD2 Oxygen Atoms^a^

	Atomic charge (*e*)	
	OD1	OD2
Original FF (CGenFF)	–0.776	–0.776
xDRESP (*bridging* mode)	–0.42 ± 0.05	–0.20 ± 0.04
xDRESP (*chelating* mode)	–0.28 ± 0.04	–0.25 ± 0.07
RESP (100% *bridging*)	–1.147	–0.405
RESP (50% *bridging*, 50% *chelating*)	–1.063	–0.489
RESP (25% *bridging*, 75% *chelating*)	–0.972	–0.580
RESP (100% *chelating*)	–0.844	–0.708

a In case of RESP fitting, the results have been obtained from model systems, imposing all the remaining charges to be the same as in the original FF.

The results from the RESP fit are reported in Table 2, where we changed the reference configurations used during the fit, *chelating vs bridging*, and we also performed multiconformation fit, where we used equal weights for both, or a 0.25–0.75 ratio, favoring the *chelating* configuration. These results represent only a limited subset of all possible charge fitting strategies, and could be extended to, e.g., allow variations of more atoms, or varying the restraint parameters for the RESP fit. However, they clearly show that large variations are present between the two conformations, and that, similarly to what was observed with the xDRESP analysis, the OD1 oxygen, which binds the MIDAS ion in both configurations, consistently presents a larger negative charge in the *bridging* coordination mode and is always more negative than the OD2 oxygen. Notably, in the xDRESP analysis, the OD1 oxygen is the only atom with such a large difference in the average xDRESP charge between the two modes among all the atoms coordinating the MIDAS ion, while the xDRESP charge of the OD2 oxygen remains similar, although its environment changes more drastically (between coordinating and noncoordinating).

These observations suggest that even if a fixed-point-charge model is potentially sufficient to describe the MIDAS ions, as its xDRESP charge is unchanged in the two configurations, the charges of its coordinating atoms are strongly influenced by the coordination mode. This may pose significant challenges for fixed-point charge FFs in accurately describing these interactions, especially when the coordination mode switches dynamically.

### 3.4 MM TI

We performed additional TI at the MM level, comparing the results of the original FF with the ones using adapted charges generated from the RESP fitting. By performing MM TI on similar windows as the QM/MM TI (Figure 4), we concluded that the two configurations are indeed minima also for the original FF, separated by a similar barrier (∼5 kcal/mol) as the one obtained from QM/MM, but the original FF favors the *chelating* mode by ∼1.6 kcal/mol. Interestingly, small changes in the charge of the two oxygen atoms can drastically change the results from the FF simulations: for instance, when using charges fitted from the *bridging* mode only, the *chelating* mode ceases to be a free energy minimum. This is the case also when using charges from the multiconfiguration fit with equal weights for the two configurations. However, when the ratio of the two is changed, assigning a larger weight to the *chelating* coordinating mode, both configurations turn out to be stable minima. Moreover, it is possible to observe a profile that is qualitatively more similar to the QM/MM TI: the two minima are still separated by a free energy barrier of ∼5 kcal/mol (*bridging* → *chelating*), and the *bridging* mode is energetically favored (by ∼5 kcal/mol). Nevertheless, the back barrier (*chelating* → *bridging*) is incorrect, and the free energy difference between the two modes is twice the QM/MM one. Additionally, the location of the minima is incorrect, especially for the *bridging* mode which is ∼0.5 Å lower for the FF-based TI profile.

**Figure 4:**
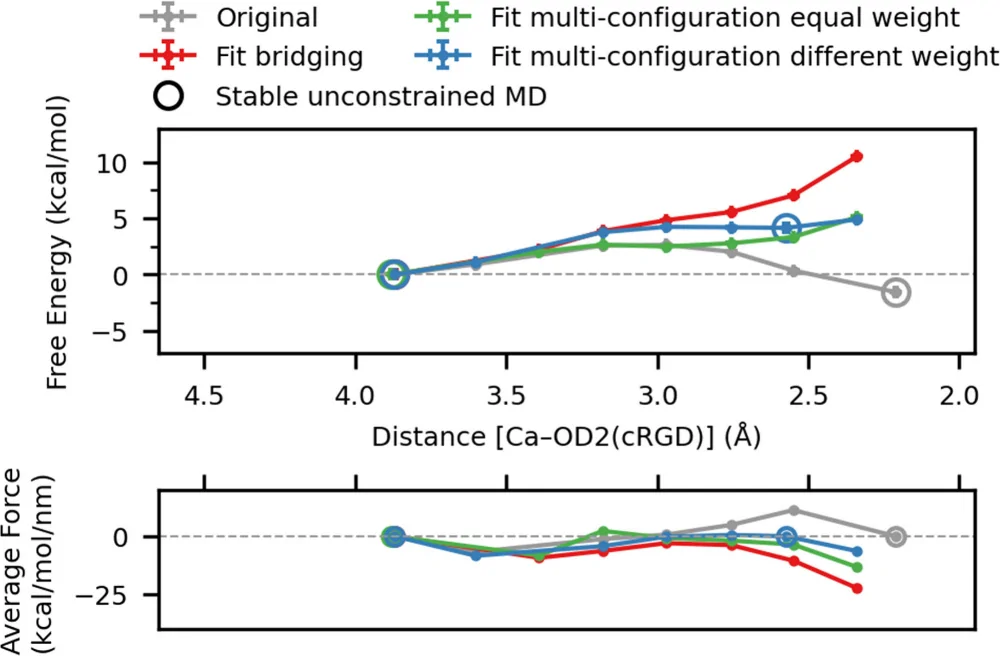
Free energy profile from MM TI (upper panel) and corresponding average constraint forces (lower panel) for different variations of the charge of the OD1 and OD2 oxygen atoms (using charges from Table 2). Unconstrained MM MD simulations have been used to add zero-force points for the stable conformations.

As mentioned in the previous section, the considered modifications of the FF charges represent only a limited subset of possible charge-fitting strategies and could be extended further. Nevertheless, the results of this section show how sensitive the metal coordination properties of a system can be to these variations, with the possibility that a conformation (e.g., the *chelating* one in this case) can become even unstable.

## 4 Conclusions and Outlook

Metal coordination modes arise from a delicate balance of electrostatics, polarization, charge redistribution, and geometric directionality. Accurately capturing this balance remains challenging for classical FFs based on fixed point charges, particularly in a dynamic environment where multiple coordination geometries may be accessible. In this work, we address this problem in the case of Ca^2+^-mediated cRGD binding to the integrin α_V_β_3_ protein. By combining classical and QM/MM MD simulations, we aim to assess whether the metal coordination modes observed in FF-based simulations correspond to physically relevant states or arise from artifacts of the FF description.

For this system, classical MD simulations using CHARMM36m/CGenFF predicted two distinct coordination modes, namely *chelating* and *bridging*, among which only the latter is supported by available experimental data.^43^ QM/MM MD simulations show that both modes correspond to free energy minima with the experimentally observed *bridging* mode being thermodynamically favored by ∼2.6 kcal/mol, and that the two are separated by a free energy barrier of ∼5.2 kcal/mol to go from the *bridging* to the *chelating* mode, and ∼2.6 kcal/mol for the opposite direction. Moreover, charge analyses of the QM/MM MD runs based on the xDRESP method as well as RESP fitting on model systems, reveal that although the effective charge of the Ca^2+^ ion remains essentially unchanged, the electronic distribution on the O atoms of the coordinating ligand is strongly affected by the coordination geometry. Consistent with this, the QM/MM description predicts systematically longer Ca–O distances (about 0.3Å) and different protein coordination numbers compared to MM MD, reinforcing that the two levels of theory yield genuinely distinct local descriptions of the metal coordination environment. Notably, small variations in the MM charges of the two coordinating oxygen atoms of the cRGD ligand are sufficient to significantly alter the free energy landscape. Moreover, it should be noted that, besides electrostatics, the short-range repulsive component of the van der Waals interaction, arising from Pauli repulsion, may also affect the free energy profile, especially the location of the equilibrium distances. In the FF, this is primarily governed by the Lennard-Jones σ or *R*_min_ parameter, and changing those values could improve the agreement with QM/MM results. Nevertheless, in this work these parameters were intentionally left unchanged to isolate the effect of the electrostatics. Together, our results demonstrate that the stability of metal coordination modes is most sensitive to electronic effects localized on the coordinating atoms, rather than on the metal ion itself, in agreement with what was previously highlighted for Mg^2+^ by Casalino et al.,^31^ suggesting that the common practice of developing site-specific ion parameters does not reflect the physical origin of these effects.

Although we only used CHARMM36m/CGenFF, which is one of the most common FF combinations in biomolecular simulations, our findings highlight the intrinsic limitations of fixed-point-charge FFs FFs in modeling metal coordination modes in biological systems, especially when asymmetric binding modes are involved. In the specific case of the MIDAS ion in integrin α_V_β_3_, this limitation is related to the assignment of identical partial charges to the two oxygen atoms of deprotonated carboxylate groups, a common feature of fixed-point-charge FFs that may hinder the accurate representation of asymmetric coordination modes. QM/MM MD offers a potential way to assess and validate FF predictions, but its computational cost limits the accessible time scales compared to classical MD. Hence, QM/MM alone cannot fully address all challenges, such as the need for long simulation times. Alternative strategies include fluctuating charge models^93–95^ and polarizable FFs,^69–72^ in which the charges of the atoms coordinating the metal can adapt to the coordination environment. To limit the computational effort, these can be performed within multilevel schemes analogous to QM/MM, i.e., where only a limited region of the system is treated with higher accuracy (e.g., with a polarizable FF). In this context, QM/MM results can be used as a reference for the parametrization of polarizable FFs for the specific metal coordination modes of interest, e.g., via a force matching approach,^96–98^ or for machine learning (ML) potentials.^99–101^ In practice, the application of such multiscale descriptions requires flexible simulation frameworks. Platforms such as MiMiC, designed to support different multilevel descriptions, can facilitate the transitions between QM/MM and, e.g., a polarizable MM/fixed-point-charge MM combined representation or ML/MM, providing an important foundation for improving the treatment of metal coordination modes in large biomolecular systems.

## Supplementary Material



## Data Availability

The data generated for this work are publicly available on Zenodo under 10.5281/zenodo.20844406, including Jupyter Notebooks to reproduce the analysis and generate the plots presented in the manuscript.

## Notes

Details on the system preparation via docking, as well as additional figures not included in the main text from MM and QM/MM molecular dynamics simulations (PDF)
