# Supplementary material for: Limitations of Fixed-Point-Charge Force Fields for Metal Coordination Modes in Proteins: A Multilevel Study of Ca2+ in Integrin αVβ3
Source: J Chem Theory Comput. 2026 Oct 28;22(21):11077–86. doi: 10.1021/acs.jctc.6c01320 (PMC13757748; doi:10.1021/acs.jctc.6c01320)
Supplement: Supplementary file 1 [file ct6c01320_si_001.pdf]

Supporting Information

Limitations of Fixed-Point-Charge Force Fields for Metal Coordination Modes in Proteins: A Multilevel Study of Ca^{2+} in Integrin $\alpha_V\beta_3$

Andrea Levy[1,†], Giulia Frigerio[2,3,†], Jules Grollier[1], Paulo Siani[2,3], Cristiana Di Valentin[2,3,*], Ursula Rothlisberger[1,*]

1. *Laboratory of Computational Chemistry and Biochemistry, École Polytechnique Fédérale de Lausanne, Lausanne, CH-1015, Switzerland*
2. *Department of Materials Science, University of Milano-Bicocca, Via R. Cozzi 55, 20125 Milan, Italy*
3. *BioNanoMedicine Center NANOMIB, University of Milano-Bicocca, 20125 Milan, Italy*

[†] These authors equally contributed to this work.

* Corresponding authors: cristiana.divalentin@unimib.it, ursula.roethlisberger@epfl.ch

Ligand Docking

The integrin $\alpha_V\beta_3$ was processed using the *Protein Preparation Wizard*,[1] while the ligand was prepared using the *LigPrep* tool and the grid generated using the *Receptor Grid Generation* tool. The rigid-receptor docking calculations were carried out at extra precision (XP) using the *Glide* software.[2–4] We obtained 6 docking poses for the cRGD in the binding pocket of integrin $\alpha_V\beta_3$, which were very close in docking score. To choose one, we used two quantitative criteria taking as reference the RGD sequence of cilengitide, a cyclic peptide that was co-crystallized with the integrin $\alpha_V\beta_3$ (PDB:1L5G).[5] The first criterion involves the values of the three geometrical criteria proposed by Kostidis et al.[6] and by Othman et al.[7] to assess the biological activity of RGD-based ligands. These three criteria are named d_1, d_2, and θ. d_1 is the distance between C_ζ and C_γ of Arg and Asp, respectively. d_2 is the distance between the C_β of both Arg and Asp. θ is the angle between C_β, C_α and C_β of Arg, Gly and Asp, respectively. To measure the deviation of the docking poses with respect to the values measured for the co-crystallized cilengitide, the following normalized Euclidian distance, named *Score*, was defined

$$Score = \sqrt{\left(\frac{d_1 - d_{1,0}}{d_{1,0}}\right)^2 + \left(\frac{d_2 - d_{2,0}}{d_{2,0}}\right)^2 + \left(\frac{\theta - \theta_0}{\theta_0}\right)^2}$$

where $d_{1,0}$, $d_{2,0}$ and θ_0 are values for the co-crystallized cilengitide.

The second criterion compares the structure of the RGD motif of each docking pose with respect to the cilengitide one using the root mean square deviation (RMSD) computed over the heavy atoms of this common RGD motif, taking as a reference the atoms of this motif in the cilengitide crystal structure.

The RMSD and the *Score* were normalized using the standard formula:

$$X_{norm} = \frac{X - X_{\min}}{X_{max} - X_{\min}}$$

where X_{norm} is the normalized value of X, X is the original value, X_{min} is the minimum value in the dataset, and X_{max} is the maximum value in the dataset. X represents either the RMSD or *Score* calculated for each docking pose normalized to a range between 0 and 1.

Based on the normalized RSMD and *Score* shown in Figure S1, pose 6 was selected and used as the starting point structure in MM MD simulations.

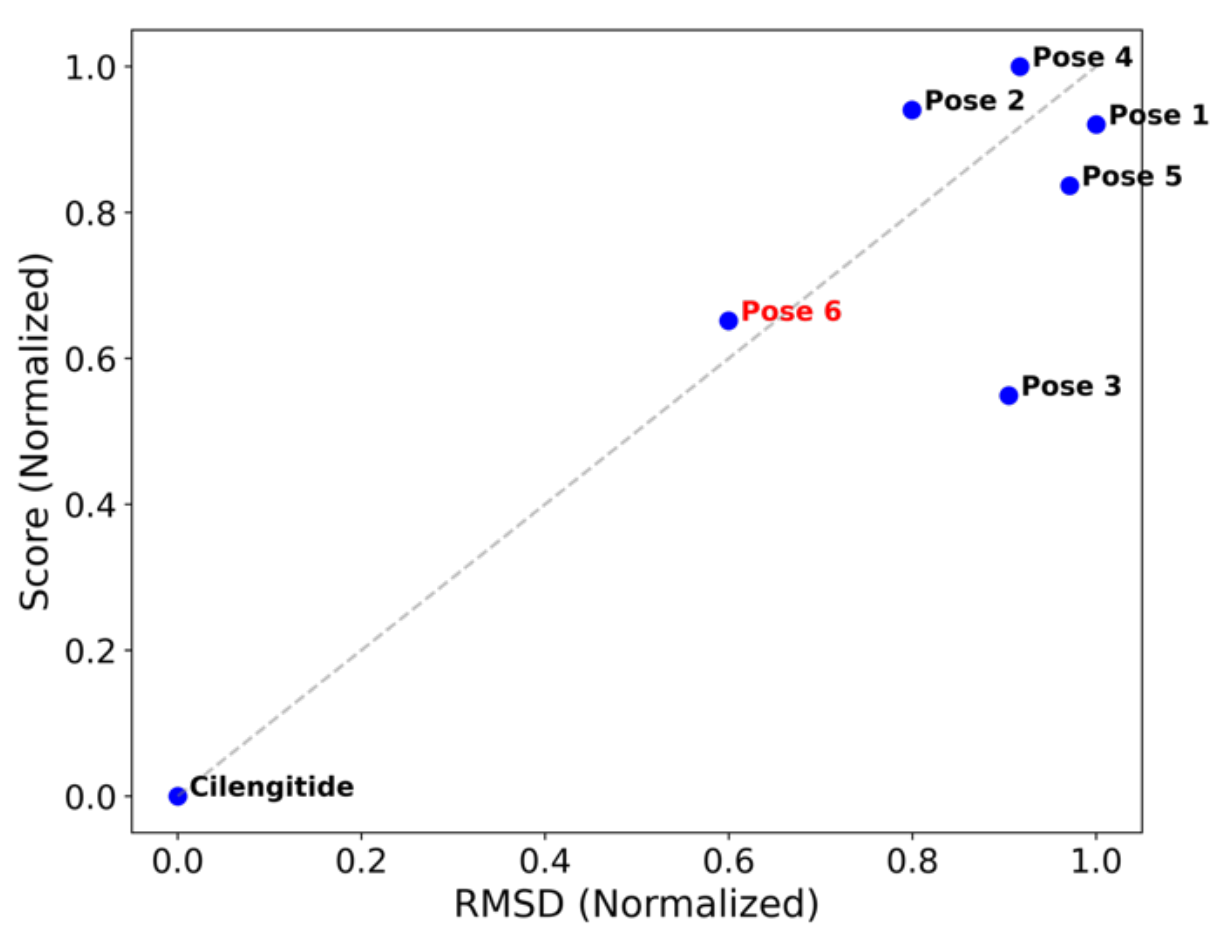

Figure S1. *Score* of the docking poses against the RMSD of the docking poses, where co-crystallized cilengitide is the reference structure.

Additional figures

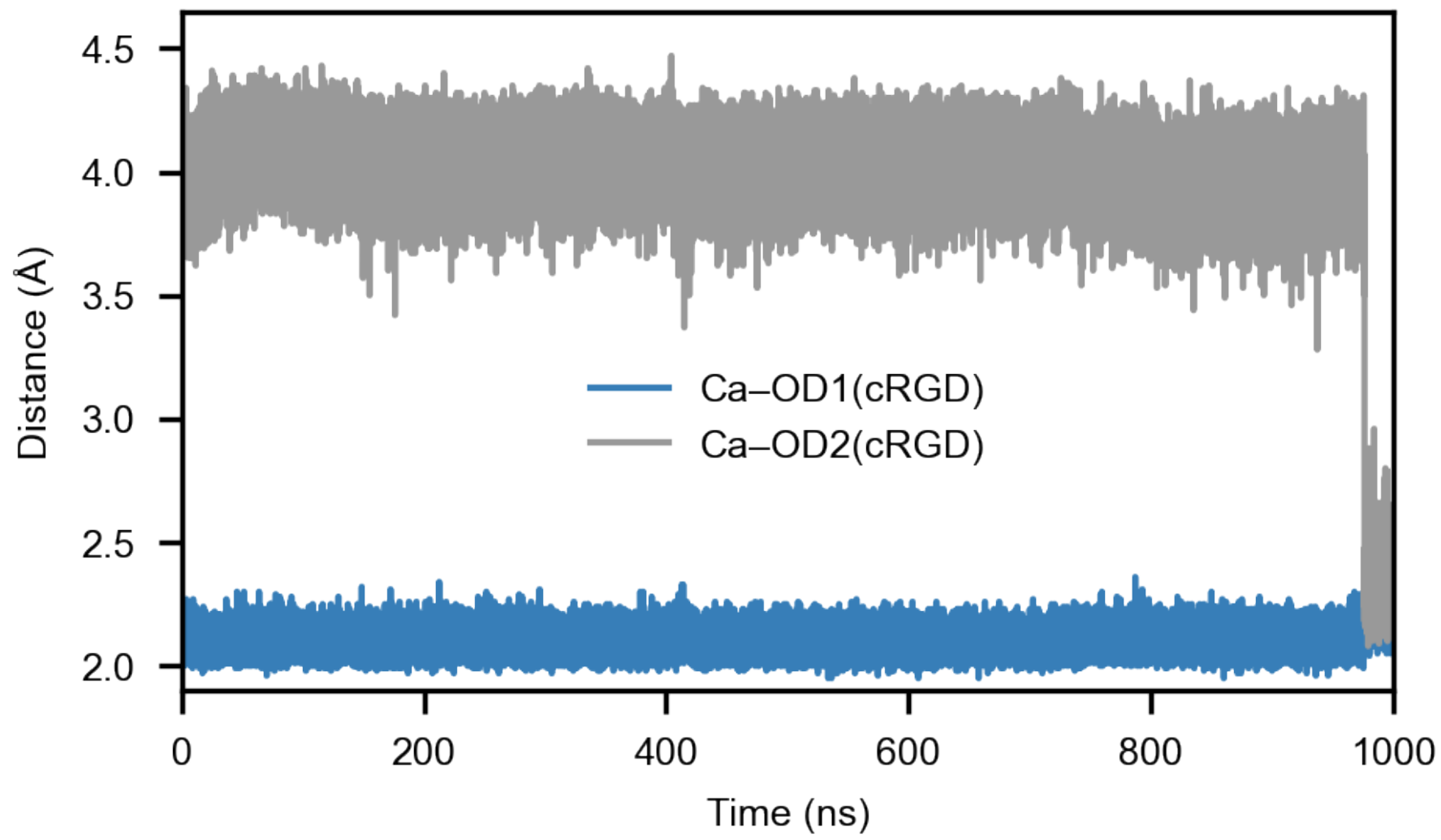

Figure S2. Ca–OD1 and Ca–OD2 distances during the 1μs MM MD.

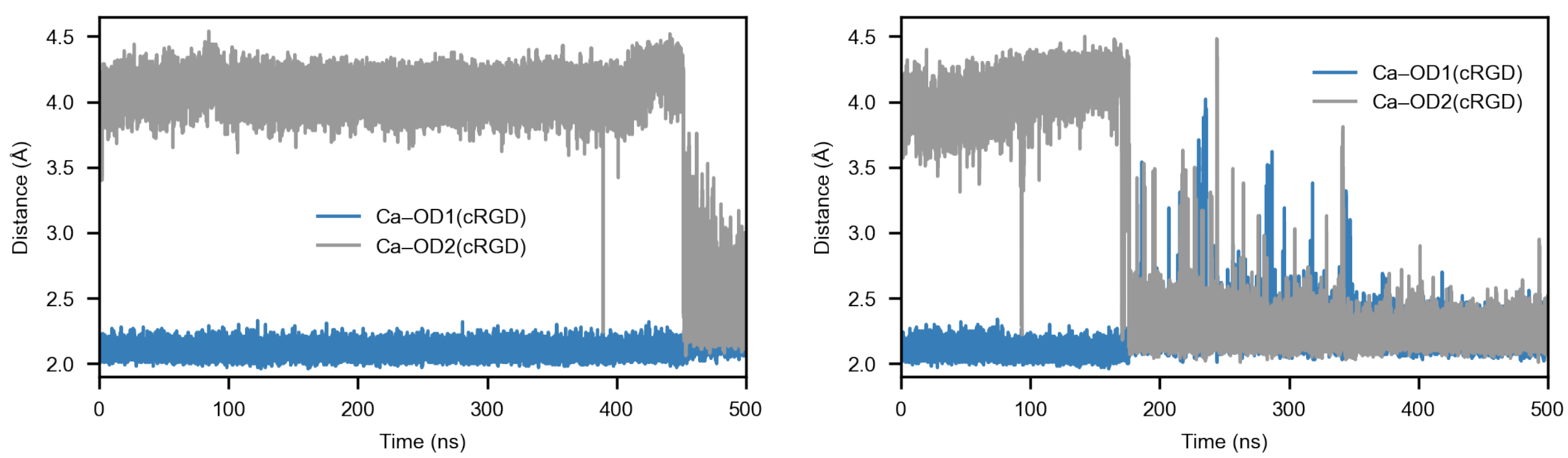

Figure S3. Ca–OD1 and Ca–OD2 distances during two 500ns replicas of the MM MD.

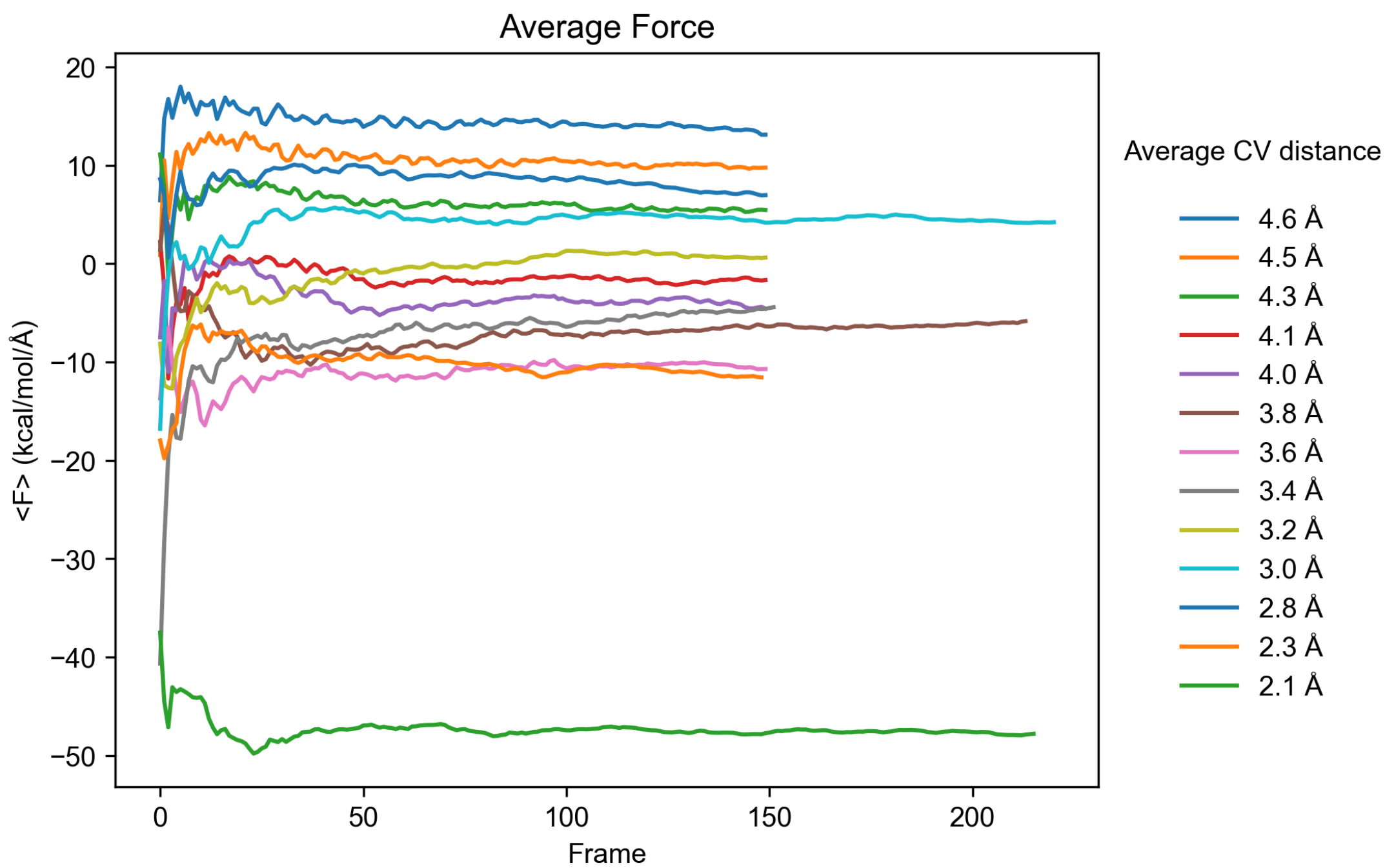

Figure S4. Convergence of the average constraint force for the QM/MM TI windows. Constraints are saved every 10 steps, i.e., every ~5fs.

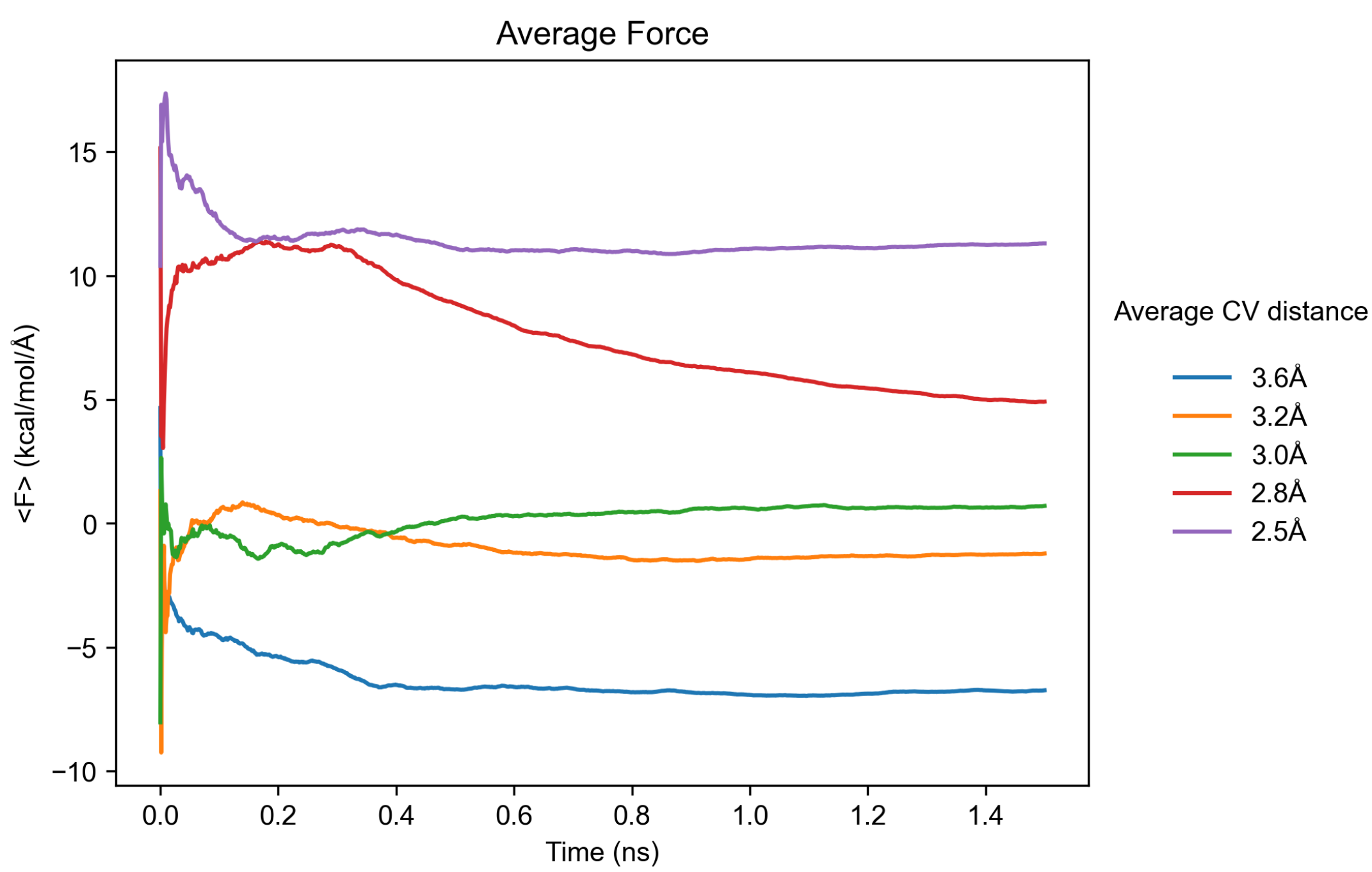

Figure S5. Convergence of the average constraint force for the MM TI windows with the original FF. Constraints are saved every 50fs.

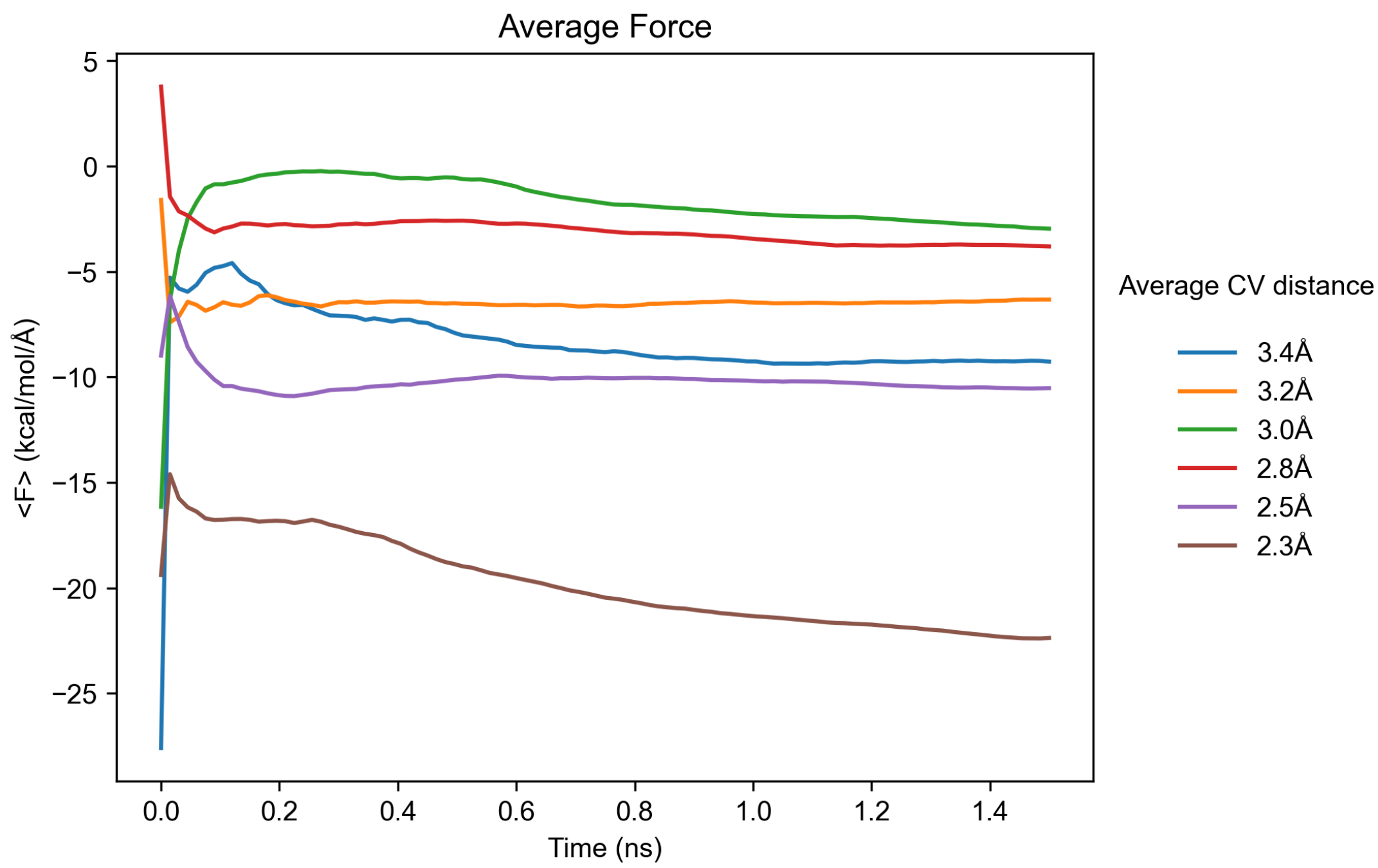

Figure S6. Convergence of the average constraint force for the MM TI windows with the modified FF (fitted to the *bridging* conformation). Constraints are saved every 50fs.

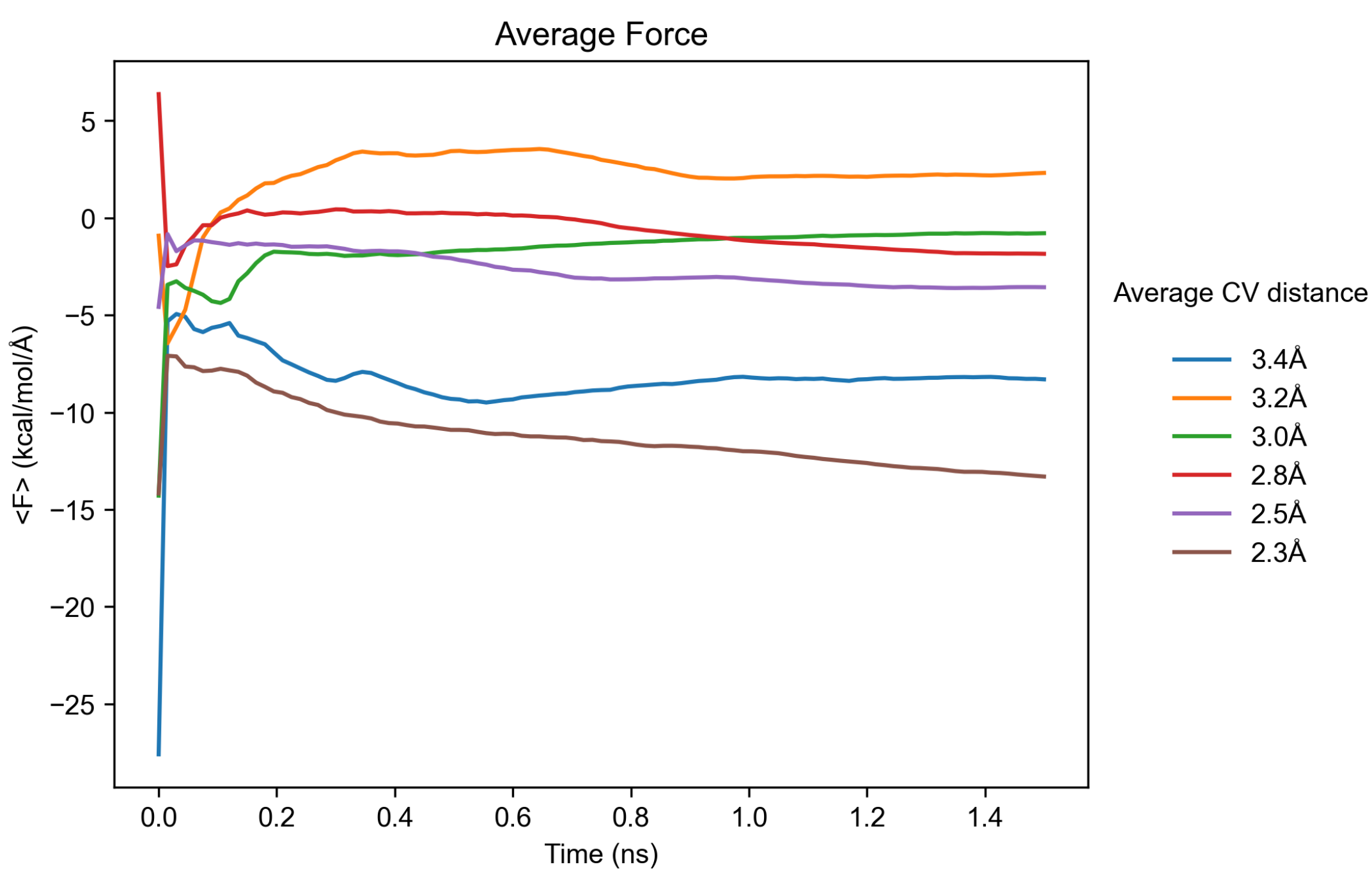

Figure S7. Convergence of the average constraint force for the MM TI windows with the modified FF (multiconfiguration fit – 50% *bridging*, 50% *chelating*). Constraints are saved every 50fs.

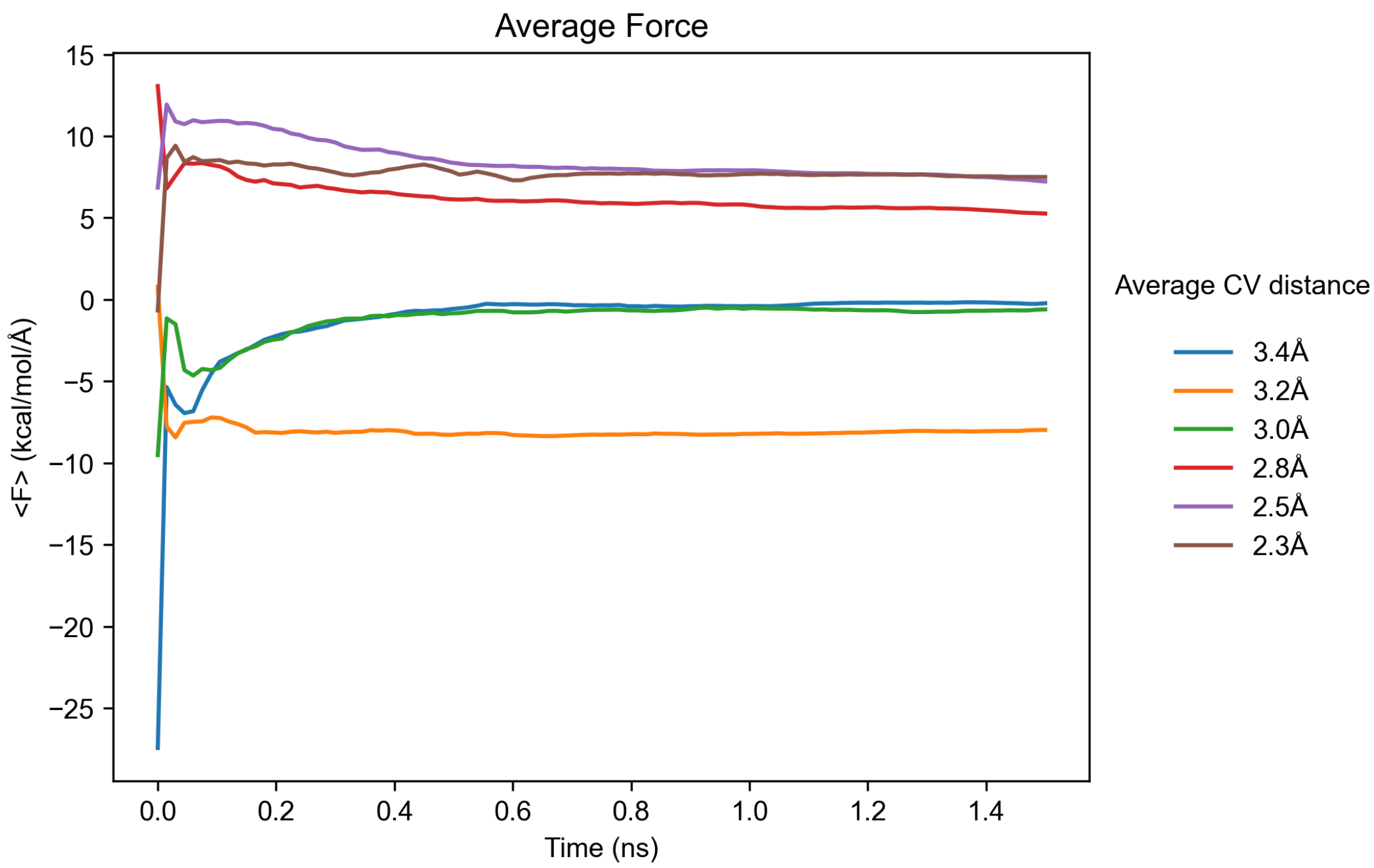

Figure S8. Convergence of the average constraint force for the MM TI windows with the modified FF (multiconfiguration fit – 25% *bridging*, 75% *chelating*). Constraints are saved every 50fs.

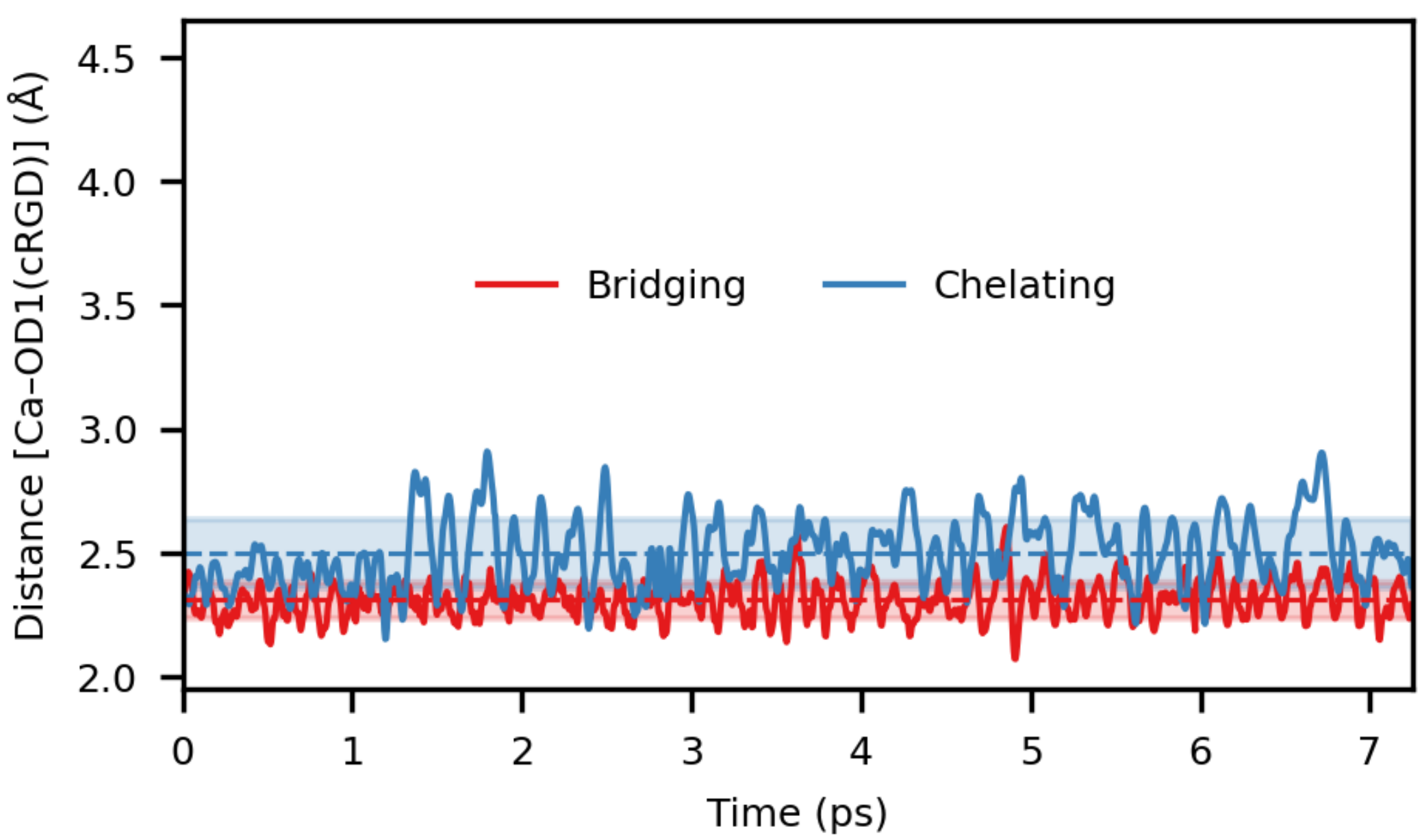

Figure S9. Ca-OD1 distance during the unconstrained QM/MM MD for the *bridging* (red) and *chelating* (blue) modes. In both cases, the average value is indicated by a dashed line, and the area of the graph within one standard deviation is highlighted with a colored bar.

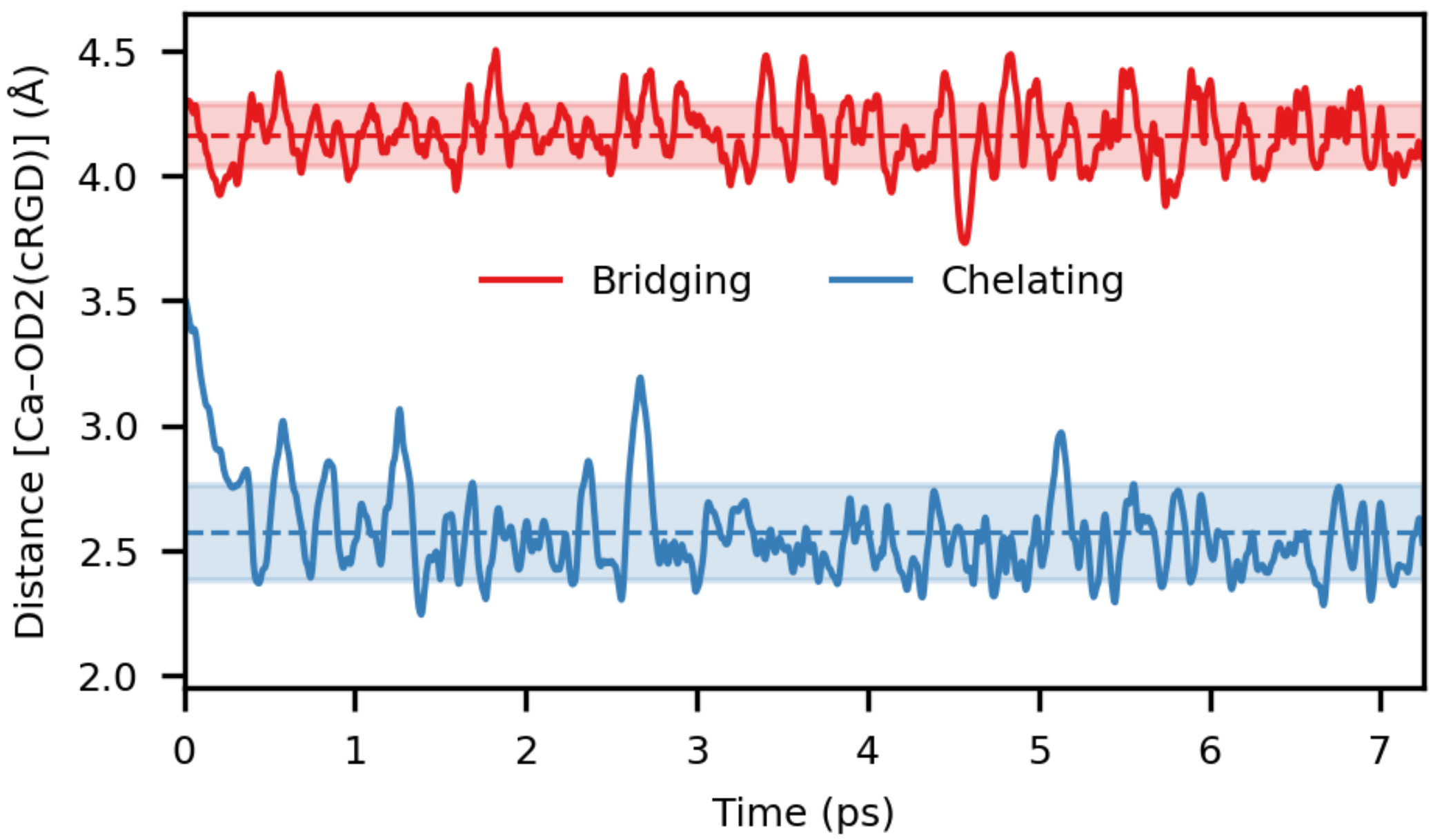

Figure S10. Ca–OD2 distance during the unconstrained QM/MM MD for the *bridging* (red) and *chelating* (blue) modes. In both cases, the average value is indicated by a dashed line, and the area of the graph within one standard deviation is highlighted with a colored bar.

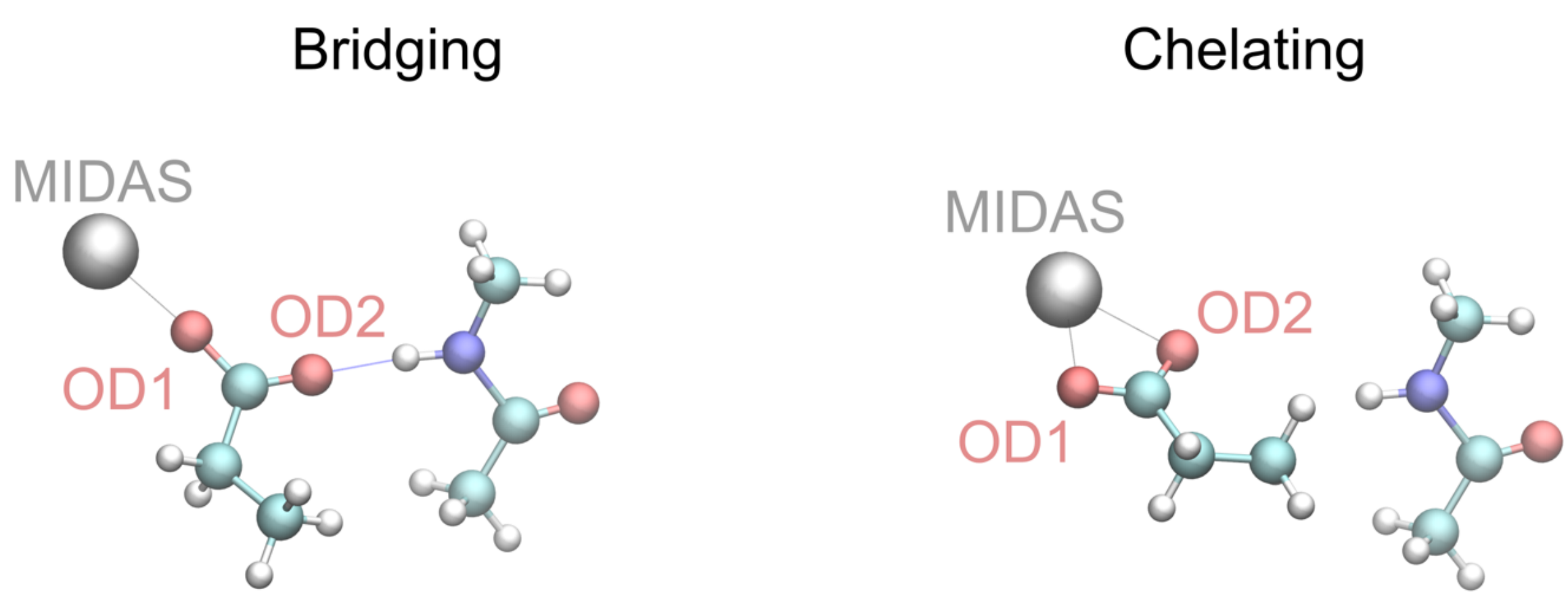

Figure S11. Model systems used for atomic charge analysis in the *bridging* (left) and *chelating* (right) modes. The reported conformations correspond to the optimized geometries at the MP2/6–31G(d) level. The Ca–OD1/2 bonds and the hydrogen bond present in the *bridging* conformation are represented in black and in blue, respectively.

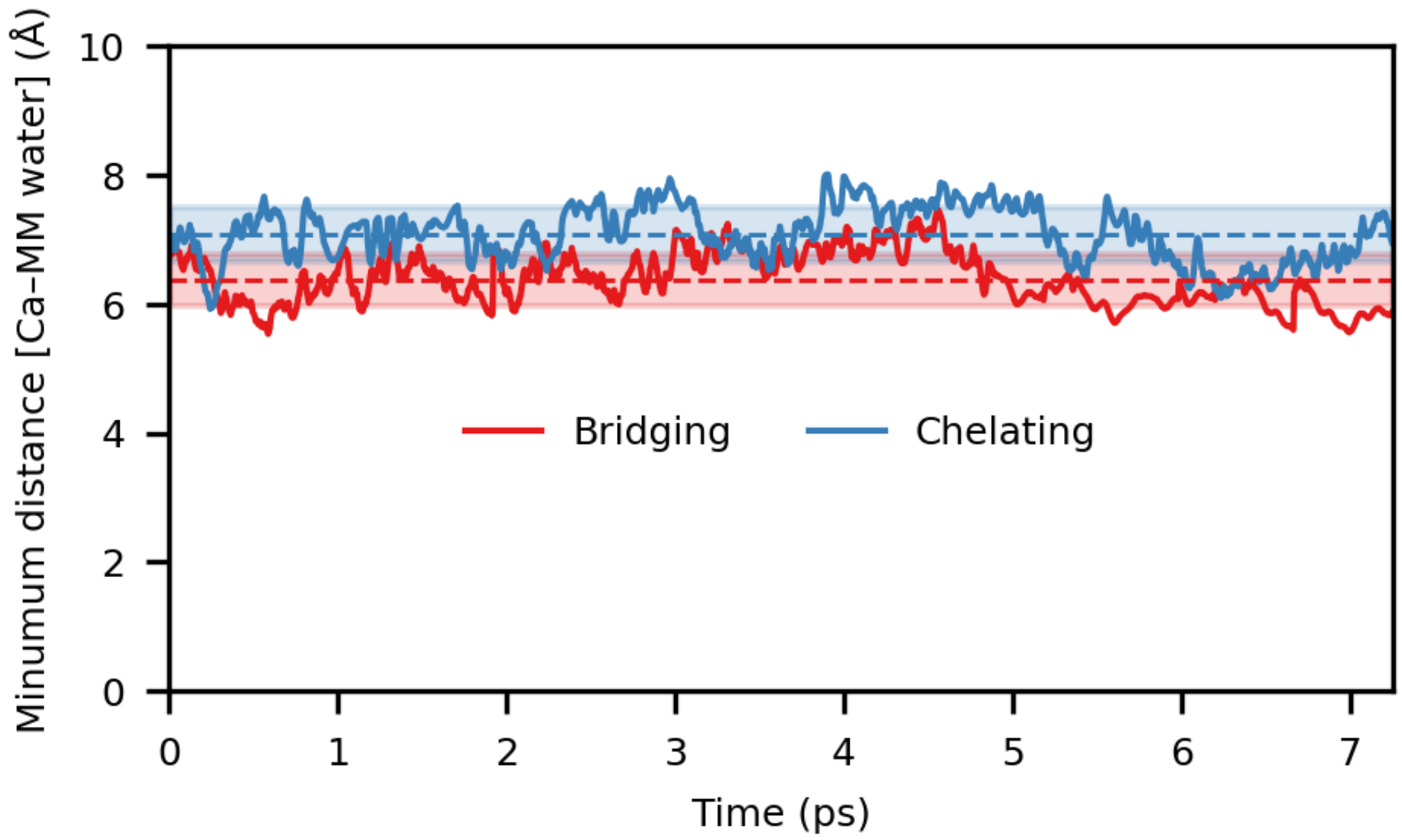

Figure S12. Minimum distance between the MIDAS ion and any MM water during the unconstrained QM/MM MD simulations in the *bridging* (red) and *chelating* (blue) conformations.

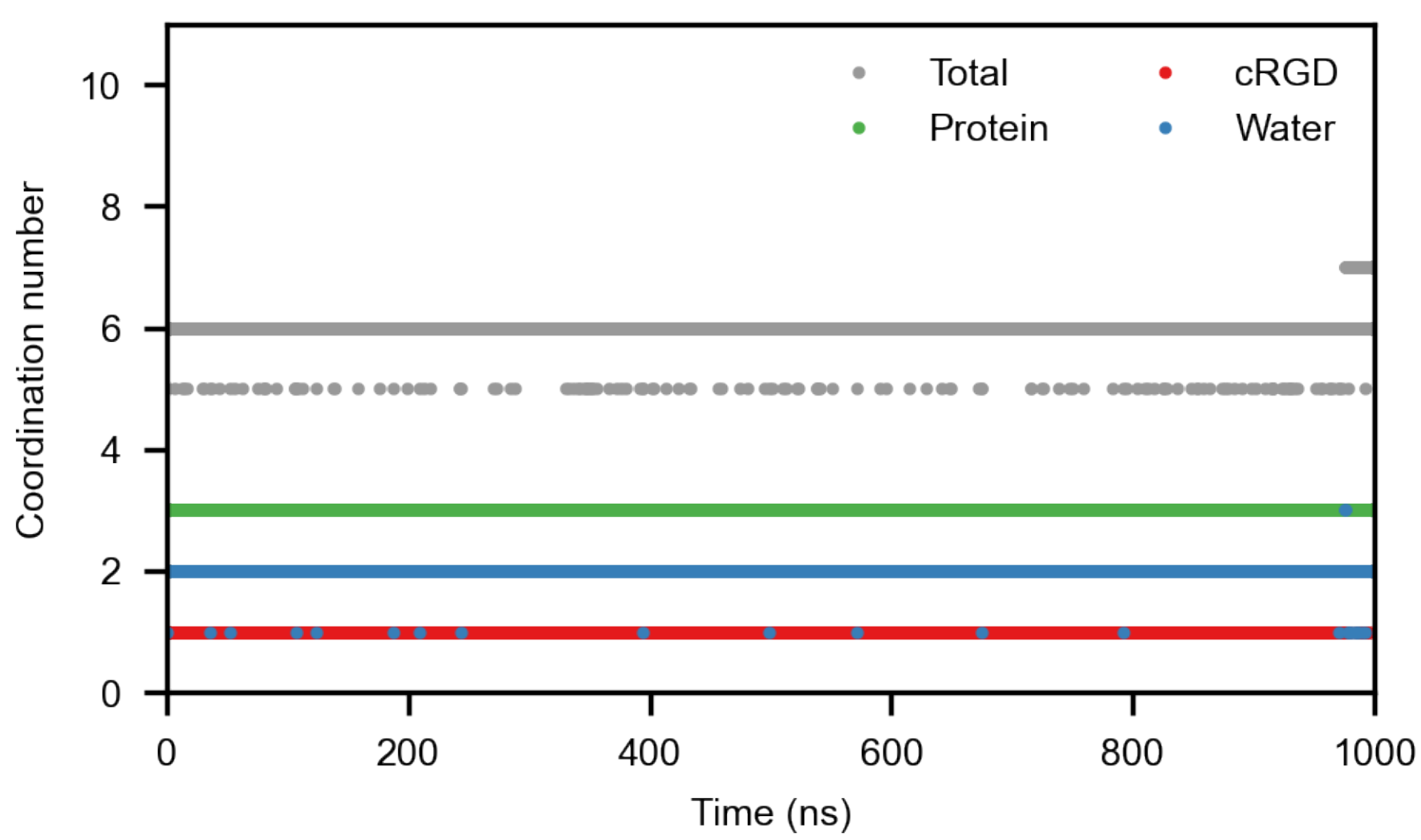

Figure S13. Coordination number of the MIDAS ion during the 1μs MM MD (cutoff 2.5 Å).

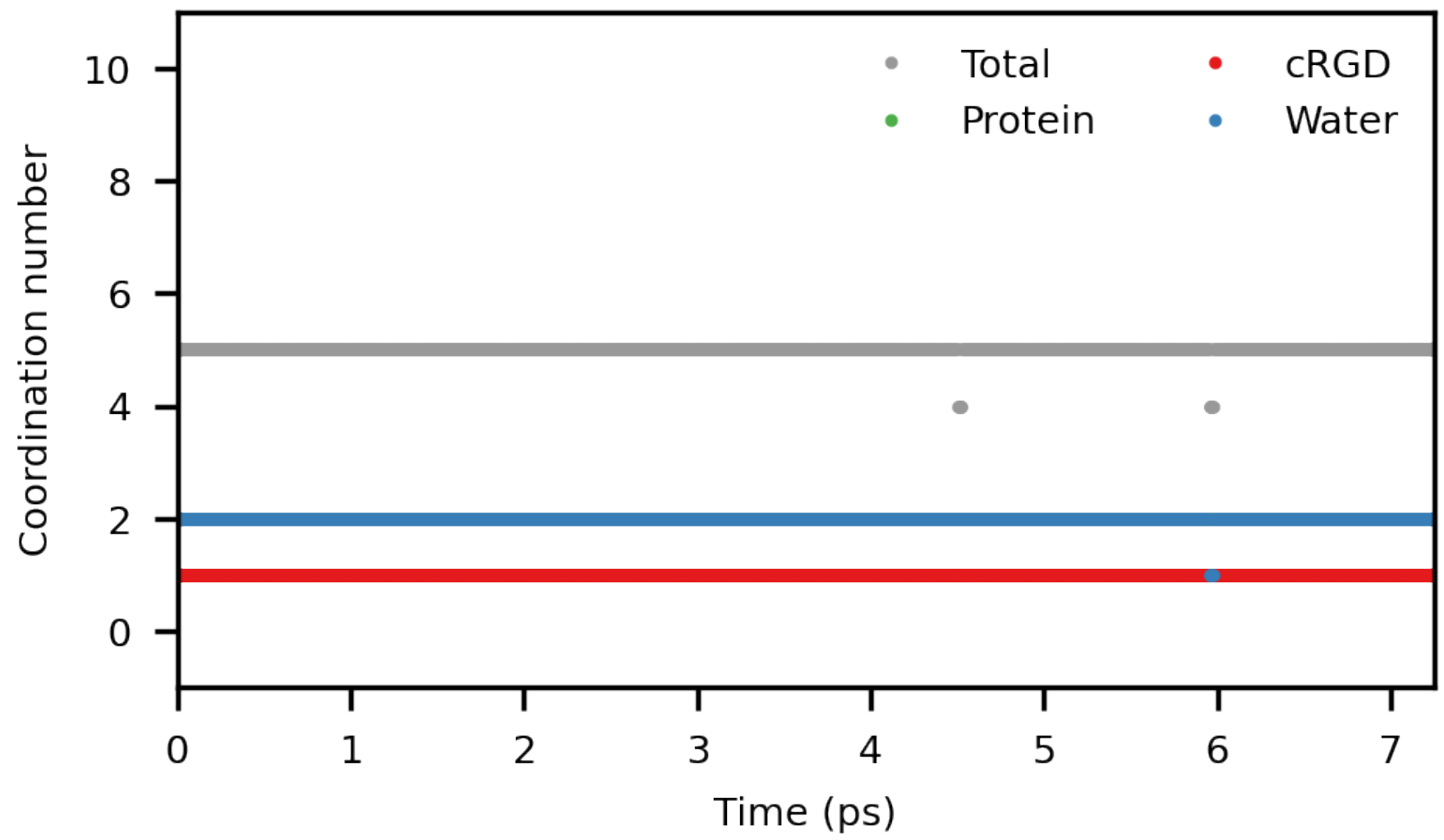

Figure S14. Coordination number of the MIDAS ion during the QM/MM MD in the (cutoff 2.8 Å) in the *bridging* mode.

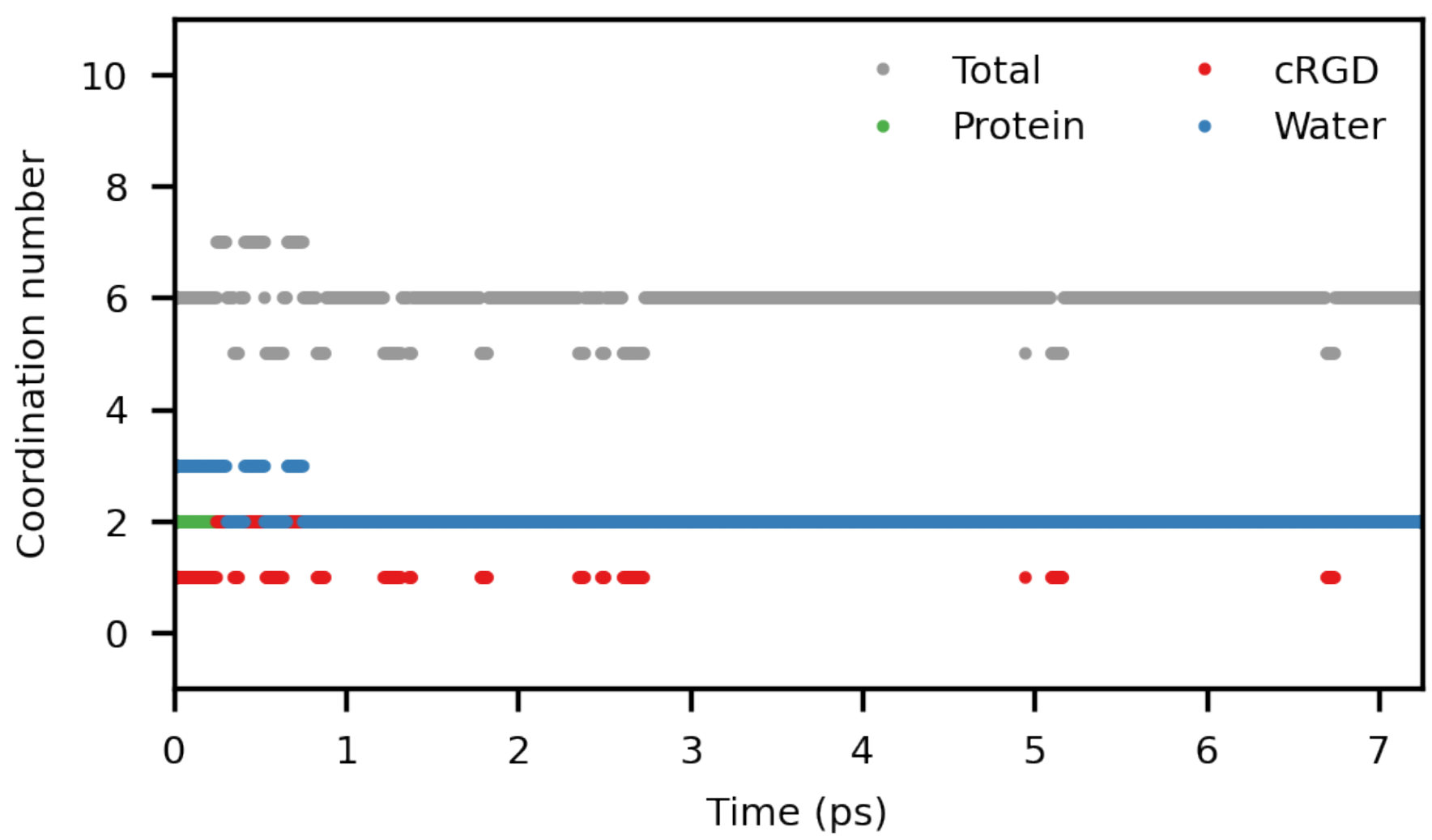

Figure S15. Coordination number of the MIDAS ion during the QM/MM MD in the (cutoff 2.8 Å) in the *chelating* mode.

References

(1) Madhavi Sastry, G.; Adzhigirey, M.; Day, T.; Annabhimoju, R.; Sherman, W. Protein and Ligand Preparation: Parameters, Protocols, and Influence on Virtual Screening Enrichments. *J. Comput. Aided. Mol. Des.* **2013**, *27* (3), 221–234. https://doi.org/10.1007/s10822-013-9644-8.

(2) Halgren, T. A.; Murphy, R. B.; Friesner, R. A.; Beard, H. S.; Frye, L. L.; Pollard, W. T.; Banks, J. L. Glide: A New Approach for Rapid, Accurate Docking and Scoring. 1. Method and Assessment of Docking Accuracy. *J. Med. Chem.* **2004**, *47* (7), 1750–1759. https://doi.org/10.1021/jm030644s.

(3) Halgren, T. A.; Murphy, R. B.; Friesner, R. A.; Beard, H. S.; Frye, L. L.; Pollard, W. T.; Banks, J. L. Glide: A New Approach for Rapid, Accurate Docking and Scoring. 2. Enrichment Factors in Database Screening. *J. Med. Chem.* **2004**, *47* (7), 1750–1759. https://doi.org/10.1021/jm030644s.

(4) Friesner, R. A.; Murphy, R. B.; Repasky, M. P.; Frye, L. L.; Greenwood, J. R.; Halgren, T. A.; Sanschagrin, P. C.; Mainz, D. T. Extra Precision Glide: Docking and Scoring Incorporating a Model of Hydrophobic Enclosure for Protein-Ligand Complexes. *J. Med. Chem.* **2006**, *49* (21), 6177–6196. https://doi.org/10.1021/jm051256o.

(5) Xiong, J.-P.; Thilo, S.; Zhang, R.; Joachimiak, A.; Frech, M.; Goodman, S. L.; Arnaout, M. A. Crystal Structure of the Extracellular Segment of Integrin Alpha Vbeta 3 in Complex with an Arg-Gly-Asp Ligand. *Science (1979).* **2002**, *296* (5565), 151–155. https://doi.org/10.1126/science.1069040.

(6) Kostidis, S.; Stavrakoudis, A.; Biris, N.; Tsoukatos, D.; Sakarellos, C.; Tsikaris, V. The Relative Orientation of the Arg and Asp Side Chains Defined by a Pseudodihedral Angle as a Key Criterion for Evaluating the Structure-Activity Relationship of RGD Peptides. *Journal of Peptide Science* **2004**, *10* (8), 494–509. https://doi.org/10.1002/psc.559.

(7) Othman, H.; Messaoud, H. Ben; Khamessi, O.; Ben-Mabrouk, H.; Ghedira, K.; Bharuthram, A.; Treurnicht, F.; Achilonu, I.; Sayed, Y.; Srairi-Abid, N. SARS-CoV-2 Spike Protein Unlikely to Bind to Integrins via the Arg-Gly-Asp (RGD) Motif of the Receptor Binding Domain: Evidence From Structural Analysis and Microscale Accelerated Molecular Dynamics. *Front. Mol. Biosci.* **2022**, *9*. https://doi.org/10.3389/fmolb.2022.834857.
